# Correction: Ecological versatility and biotechnological promise: Comprehensive characterization of the isolated thermophilic *Bacillus* strains

**DOI:** 10.1371/journal.pone.0336510

**Published:** 2025-11-07

**Authors:** Hazem Aqel, Husni Farah, Afnan Al-Hunaiti

There is an error in affiliation 1 for author Hazem Aqel. The correct affiliation 1 is: Basic Medical Sciences Department, College of Medicine, Al-Balqa Applied University, Salt, Jordan.
