# Supplementary material for: Ecological versatility and biotechnological promise: Comprehensive characterization of the isolated thermophilic Bacillus strains
Source: PLoS One. 2024 Apr 18;19(4):e0297217. doi: 10.1371/journal.pone.0297217 (PMC11025799; doi:10.1371/journal.pone.0297217)

**S7 Fig. Cell morphology of TBS35 using Gram-stain, spores were subterminal. Bacteria cell morphology (A) at 60^o^C; and (B) bacteria grew at 73^o^C.**


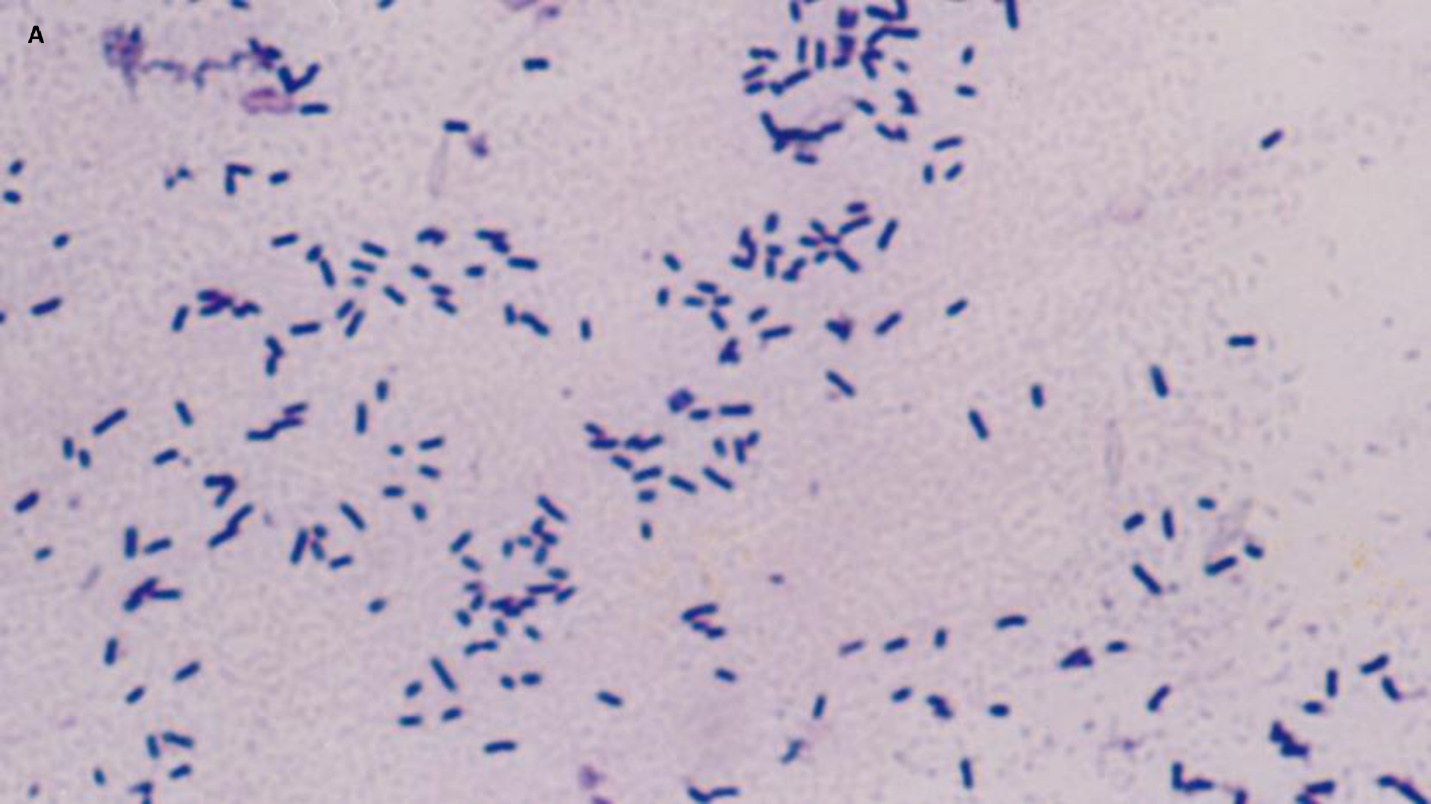


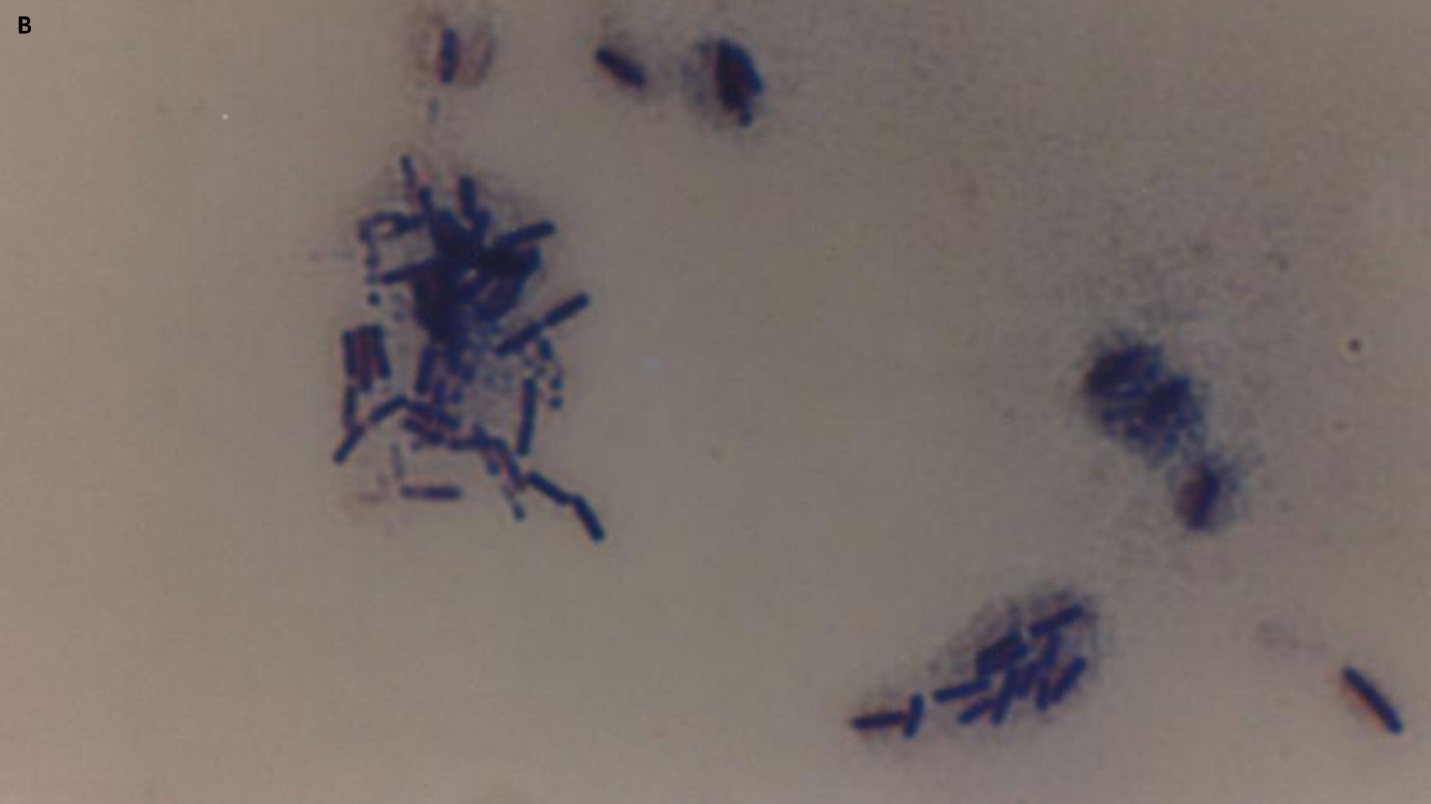

Supplement: S7 Fig — Bacteria cell morphology (A) at 60°C; and (B) bacteria grew at 73°C. (DOCX) [file pone.0297217.s007.docx]
