# Supplementary material for: Ecological versatility and biotechnological promise: Comprehensive characterization of the isolated thermophilic Bacillus strains
Source: PLoS One. 2024 Apr 18;19(4):e0297217. doi: 10.1371/journal.pone.0297217 (PMC11025799; doi:10.1371/journal.pone.0297217)
Supplement: S1 Table — (DOCX) [file pone.0297217.s011.docx]

S1 Table. Cell morphology of isolated thermophilic Bacillus strains at optimum and maximum temperatures.

| Isolated Thermophilic *Bacillus* strains | Temperature for bacterial growth | | Bacterial cell morphology | |
| --- | --- | --- | --- | --- |
|  | **Optimum** | **Maximum** | **Optimum** | **Maximum** |
| TBS4 | 50^o^C | 73^o^C | Thin rods with subterminal spore | Very thick rods |
| TBS5 | 58^o^C | 73^o^C | Short thin rods with central and subterminal spore | Long thick rod |
| TBS6 | 51^o^C | 73^o^C | Short thick rods with subterminal spore | Chain short thick rods |
| TBS25 | 54^o^C | 73^o^C | Short thick rods | Long thick rods with subterminal spore |
| TBS26 | 51^o^C | 73^o^C | Short rods with central and subterminal spore | Long thick rods with subterminal spore |
| TBS34 | 54^o^C | 73^o^C | Long thick rods with subterminal spore | Long thin rods |
| TBS35 | 60^o^C | 73^o^C | Very short rods | Short thick rods |
| TBS40 | 60^o^C | 73^o^C | Very short rods | Very short rods |
| TBS45 | 55^o^C | 73^o^C | Short thin rods with terminal spore | Short thick rods |
| TBS55 | 52^o^C | 73^o^C | Short thick rods with central spore | Short thick rods |
