# Supplementary material for: Ecological versatility and biotechnological promise: Comprehensive characterization of the isolated thermophilic Bacillus strains
Source: PLoS One. 2024 Apr 18;19(4):e0297217. doi: 10.1371/journal.pone.0297217 (PMC11025799; doi:10.1371/journal.pone.0297217)
Supplement: Attachment — Submitted filename: Response to Reviewers.docx [file pone.0297217.s012.docx]

**Reviewer #1,**

Thank you for your comments and suggestions on our manuscript. Based on your feedback, we have revised and corrected the abstract to better reflect the scope and findings of our study. The modified abstract now clearly outlines the focus on the adaptability and physiological diversity of isolated thermophilic *Bacillus* species, emphasizing their ecological roles and potential industrial applications.

We have expanded our investigation of these species' capacity to thrive in extreme conditions by detailing their environmental tolerances and adaptations at the metabolic and genetic levels. The abstract now explicitly states the primary objective of evaluating the suitability of these species for biotechnological applications, considering their resilience in harsh environments.

Furthermore, we have included a more comprehensive description of our comparative analysis of the environmental adaptability parameters for various *Bacillus* species. This encompasses growth temperature ranges, pH tolerance, oxygen requirements, carbohydrate fermentation patterns, colony morphology, enzymatic activities, and genetic properties. We have also specified the controlled laboratory experiments that provided the data for our analysis.

Key findings have been highlighted more prominently, such as the species' ability to endure temperatures as high as 73°C, their preference for slightly alkaline conditions, and their classification into aerobic, anaerobic, and facultative aerobic categories based on oxygen requirement tests. Genetic analysis and enzymatic profiles have also been elaborated to showcase the adaptability and diversity of these species under various environmental conditions.

Overall, the revised abstract more accurately and comprehensively conveys the significance of our findings and the potential of thermophilic *Bacillus* species in biotechnological applications. We believe these changes address your concerns and enhance the clarity and impact of the abstract.

Thank you for your constructive feedback, which has been invaluable in improving our manuscript.

Sincerely,

Hazem

**Reviewer #2,**

Thank you for your invaluable feedback and insights. We have addressed the concerns you raised in your review, particularly regarding the molecular characterization of the *Bacillus* strains and the significance and usefulness of our study.

**Molecular Characterization:**

In response to your suggestion, we have conducted additional molecular analyses to characterize the bacterial species more definitively. This involved using %G+C content and PCR-based methods, which have enriched our understanding of the identity of the *Bacillus* strains. The results of these analyses are now included in the revised manuscript, providing a more robust molecular characterization of the isolated strains.

**Significance and usefulness of the study:**

We have clarified the significance of our study to the wider scientific community. Our findings contribute new knowledge in the field of microbiology, particularly in understanding the adaptability and physiological diversity of thermophilic *Bacillus* species. The potential applications of these findings in biotechnology and other relevant fields have been emphasized, including the use of these bacteria in bioremediation, biofuel production, and pharmaceutical manufacturing.

**Practical Applications of the data:**

The revised manuscript now includes a detailed explanation of how our research findings can be applied in various biotechnological applications. We elucidate the potential uses of these *Bacillus* strains, considering their ability to withstand extreme conditions, their diverse metabolic capabilities, and the genetic adaptability they exhibit. This section aims to bridge the gap between our fundamental research and its practical applications, highlighting the industrial relevance of our findings.

**Results and Discussion:**

The revised Results and Discussion sections incorporate a comprehensive analysis of DNA concentration, 16s rRNA gene length, and G+C content. These sections provide insights into the genetic diversity of the *Bacillus* species studied, emphasizing their adaptability and potential in various applications. The discussion now includes a comparative analysis of the environmental adaptability parameters, providing a deeper understanding of the physiological and genetic diversity of these species.

**Conclusions:**

The conclusions have been updated to reflect the comprehensive nature of our study. They emphasize the implications of our findings in understanding the roles of thermophilic *Bacillus* species in natural ecosystems and their potential applications in industrial processes, bioremediation, and biotechnology. The diversity in adaptations observed in these bacteria underscores their ecological versatility and potential as robust candidates for a variety of biotechnological applications.

We believe these revisions and additions address your concerns and significantly enhance the quality and impact of our manuscript. Your feedback has been instrumental in guiding these improvements, and we are grateful for your thorough and constructive review.

Sincerely,

Hazem

Dear Dr. Nasim,

**Subject:** Submission of Revised Manuscript to PLOS ONE

I am writing to submit the revised version of our manuscript [Ecological Versatility and Biotechnological Promise: Comprehensive Characterization of The Isolated Thermophilic *Bacillus* Strains] for consideration in PLOS ONE. We have carefully reviewed and addressed the comments and suggestions provided by the reviewers. Enclosed with this letter are the following documents as per the journal's requirements:

1. **Marked-up copy of the manuscript:** This version highlights all the changes made in response to the reviewers' comments. Changes include textual revisions, additional data, and methodological clarifications. We have used track changes for easy identification of the modifications.
2. **Unmarked version of the revised paper:** This is a clean copy of the revised manuscript, incorporating all the changes without the markup. It is formatted according to the guidelines of PLOS ONE and is ready for publication.

Dear [Reviewer #1] and [Reviewer #2],

Thank you for your constructive comments and suggestions regarding our manuscript [Ecological Versatility and Biotechnological Promise: Comprehensive Characterization of The Isolated Thermophilic *Bacillus* Strains]. Your feedback has greatly contributed to enhancing the quality and clarity of our research. Below, we address each point raised and explain how we have incorporated your feedback into the revised manuscript.

**Response to Reviewer #1**

1. **Point about data supporting findings:** We have revised the abstract and relevant sections to include specific data points and summaries that support our claims. These changes provide clearer evidence of the adaptability and potential applications of *Bacillus* strains.
2. **Clarification of objectives:** The objectives of the study have been explicitly stated in the abstract and introduction, providing a clear outline of our research aims.

**Respond to Reviewer #2**

1. **Significance of the study:** We have expanded the discussion section to elaborate on how our findings contribute new knowledge to the scientific community, especially in the context of biotechnology and microbial ecology.
2. **Practical applications:** We have included a detailed explanation of the potential biotechnological applications of the *Bacillus* strains in applied research, emphasizing their relevance in industrial processes and environmental conservation.

**General Formatting and submission guidelines**

We have ensured that the manuscript adheres to the PLOS ONE formatting and submission guidelines. All figures and tables are formatted as per the journal's requirements, and references have been updated accordingly.

We believe that the revisions made have significantly strengthened the manuscript, making it a valuable contribution to PLOS ONE. We appreciate the opportunity to revise our manuscript and thank the reviewers and the editor for their insightful and constructive feedback.

Sincerely,

Hazem
